# Continuing a search for a diagnosis: the impact of adolescence and family dynamics

**DOI:** 10.1186/s13023-022-02598-x

**Published:** 2023-01-09

**Authors:** Ilana M. Miller, Beverly M. Yashar, Undiagnosed Disease Network, Maria T. Acosta, Margaret Adam, David R. Adams, Pankaj B. Agrawal, Justin Alvey, Laura Amendola, Ashley Andrews, Euan A. Ashley, Mahshid S. Azamian, Carlos A. Bacino, Guney Bademci, Eva Baker, Ashok Balasubramanyam, Dustin Baldridge, Jim Bale, Michael Bamshad, Deborah Barbouth, Pinar Bayrak-Toydemir, Anita Beck, Alan H. Beggs, Edward Behrens, Gill Bejerano, Hugo J. Bellen, Jimmy Bennett, Beverly Berg-Rood, Jonathan A. Bernstein, Gerard T. Berry, Anna Bican, Stephanie Bivona, Elizabeth Blue, John Bohnsack, Carsten Bonnenmann, Devon Bonner, Lorenzo Botto, Brenna Boyd, Lauren C. Briere, Elly Brokamp, Gabrielle Brown, Elizabeth A. Burke, Lindsay C. Burrage, Manish J. Butte, Peter Byers, William E. Byrd, John Carey, Olveen Carrasquillo, Ta Chen Peter Chang, Sirisak Chanprasert, Hsiao-Tuan Chao, Gary D. Clark, Terra R. Coakley, Laurel A. Cobban, Joy D. Cogan, Matthew Coggins, F. Sessions Cole, Heather A. Colley, Cynthia M. Cooper, Heidi Cope, William J. Craigen, Andrew B. Crouse, Michael Cunningham, Precilla D’Souza, Hongzheng Dai, Surendra Dasari, Joie Davis, Jyoti G. Dayal, Esteban C. Dell’Angelica, Katrina Dipple, Daniel Doherty, Naghmeh Dorrani, Argenia L. Doss, Emilie D. Douine, David D. Draper, Laura Duncan, Dawn Earl, David J. Eckstein, Lisa T. Emrick, Christine M. Eng, Cecilia Esteves, Marni Falk, Liliana Fernandez, Carlos Ferreira, Elizabeth L. Fieg, Laurie C. Findley, Paul G. Fisher, Brent L. Fogel, Irman Forghani, William A. Gahl, Ian Glass, Bernadette Gochuico, Rena A. Godfrey, Katie Golden-Grant, Madison P. Goldrich, David B. Goldstein, Alana Grajewski, Catherine A. Groden, Irma Gutierrez, Sihoun Hahn, Rizwan Hamid, Kelly Hassey, Nichole Hayes, Frances High, Anne Hing, Fuki M. Hisama, Ingrid A. Holm, Jason Hom, Martha Horike-Pyne, Yong Huang, Alden Huang, Laryssa Huryn, Rosario Isasi, Kosuke Izumi, Fariha Jamal, Gail P. Jarvik, Jeffrey Jarvik, Suman Jayadev, Lefkothea Karaviti, Jennifer Kennedy, Shamika Ketkar, Dana Kiley, Gonench Kilich, Shilpa N. Kobren, Isaac S. Kohane, Jennefer N. Kohler, Susan Korrick, Mary Kozuira, Deborah Krakow, Donna M. Krasnewich, Elijah Kravets, Joel B. Krier, Seema R. Lalani, Byron Lam, Christina Lam, Grace L. LaMoure, Brendan C. Lanpher, Ian R. Lanza, Lea Latham, Kimberly LeBlanc, Brendan H. Lee, Hane Lee, Roy Levitt, Richard A. Lewis, Sharyn A. Lincoln, Pengfei Liu, Xue Zhong Liu, Nicola Longo, Sandra K. Loo, Joseph Loscalzo, Richard L. Maas, John MacDowall, Ellen F. Macnamara, Calum A. MacRae, Valerie V. Maduro, Rachel Mahoney, Bryan C. Mak, May Christine V. Malicdan, Laura A. Mamounas, Teri A. Manolio, Rong Mao, Kenneth Maravilla, Thomas C. Markello, Ronit Marom, Gabor Marth, Beth A. Martin, Martin G. Martin, Julian A. Martfnez-Agosto, Shruti Marwaha, Jacob McCauley, Allyn McConkie-Rosell, Alexa T. McCray, Elisabeth McGee, Heather Mefford, J. Lawrence Merritt, Matthew Might, Ghayda Mirzaa, Eva Morava, Paolo M. Moretti, Paolo Moretti, Deborah Mosbrook-Davis, John J. Mulvihill, Mariko Nakano-Okuno, Avi Nath, Stanley F. Nelson, John H. Newman, Sarah K. Nicholas, Deborah Nickerson, Shirley Nieves-Rodriguez, Donna Novacic, Devin Oglesbee, James P. Orengo, Laura Pace, Stephen Pak, J. Carl Pallais, Christina G. S. Palmer, Jeanette C. Papp, Neil H. Parker, John A. Phillips, Jennifer E. Posey, Lorraine Potocki, Bradley Power, Barbara N. Pusey, Aaron Quinlan, Archana N. Raja, Deepak A. Rao, Anna Raper, Wendy Raskind, Genecee Renteria, Chloe M. Reuter, Lynette Rives, Amy K. Robertson, Lance H. Rodan, Jill A. Rosenfeld, Natalie Rosenwasser, Francis Rossignol, Maura Ruzhnikov, Ralph Sacco, Jacinda B. Sampson, Mario Saporta, Judy Schaechter, Timothy Schedl, Kelly Schoch, Daryl A. Scott, C. Ron Scott, Vandana Shashi, Jimann Shin, Rebecca H. Signer, Edwin K. Silverman, Janet S. Sinsheimer, Kathy Sisco, Edward C. Smith, Kevin S. Smith, Emily Solem, Lilianna Solnica-Krezel, Ben Solomon, Rebecca C. Spillmann, Joan M. Stoler, Kathleen Sullivan, Jennifer A. Sullivan, Angela Sun, Shirley Sutton, David A. Sweetser, Virginia Sybert, Holly K. Tabor, Queenie K.-G. Tan, Amelia L. M. Tan, Mustafa Tekin, Fred Telischi, Willa Thorson, Audrey Thurm, Cynthia J. Tifft, Camilo Toro, Alyssa A. Tran, Brianna M. Tucker, Tiina K. Urv, Adeline Vanderver, Matt Velinder, Dave Viskochil, Tiphanie P. Vogel, Colleen E. Wahl, Melissa Walker, Stephanie Wallace, Nicole M. Walley, Chris A. Walsh, Jennifer Wambach, Jijun Wan, Lee-kai Wang, Michael F. Wangler, Patricia A. Ward, Daniel Wegner, Monika Weisz Hubshman, Mark Wener, Tara Wenger, Katherine Wesseling Perry, Monte Westerfield, Matthew T. Wheeler, Jordan Whitlock, Lynne A. Wolfe, Jeremy D. Woods, Kim Worley, Shinya Yamamoto, John Yang, Muhammad Yousef, Diane B. Zastrow, Wadih Zein, Zhe Zhang, Chunli Zhao, Stephan Zuchner, Ellen F. Macnamara

**Affiliations:** 1 Children’s National Medical Center, Rare Disease Institute, 7125 13th Place NW, DC 20012 Washington, USA; 2 Department of Human Genetics, University of Michigan, 4909 Buhl Building, Catherine St, Ann Arbor, MI 48109 USA; 3 Common Fund, Office of the Director, NIH, Bethesda, MD USA; 4 National Institutes of Health Undiagnosed Diseases Program, Common Fund, Office of the Director, NIH, Bethesda, MD USA

## Abstract

The “diagnostic odyssey” describes the process those with undiagnosed conditions undergo to identify a diagnosis. Throughout this process, families of children with undiagnosed conditions have multiple opportunities to decide whether to continue or stop their search for a diagnosis and accept the lack of a diagnostic label. Previous studies identified factors motivating a family to begin searching, but there is limited information about the decision-making process in a prolonged search and how the affected child impacts a family’s decision. This study aimed to understand how families of children with undiagnosed diseases decide whether to continue to pursue a diagnosis after standard clinical testing has failed. Parents who applied to the Undiagnosed Disease Network (UDN) at the National Institutes of Health (NIH) were recruited to participate in semi-structured interviews. The 2015 Supportive Care Needs model by Pelenstov, which defines critical needs in families with rare/undiagnosed diseases, provided a framework for interview guide development and transcript analysis (Pelentsov et al in Disabil Health J 8(4):475–491, 2015. 10.1016/J.DHJO.2015.03.009). A deductive, iterative coding approach was used to identify common unifying themes. Fourteen parents from 13 families were interviewed. The average child’s age was 11 years (range 3–18) and an average 63% of their life had been spent searching for a diagnosis. Our analysis found that alignment or misalignment of parent and child needs impact the trajectory of the diagnostic search. When needs and desires align, reevaluation of a decision to pursue a diagnosis is limited. However, when there is conflict between parent and child desires, there is reevaluation, and often a pause, in the search. This tension is exacerbated when children are adolescents and attempting to balance their dependence on parents for medical care with a natural desire for independence. Our results provide novel insights into the roles of adolescents in the diagnostic odyssey. The tension between desired and realistic developmental outcomes for parents and adolescents impacts if, and how, the search for a diagnosis progresses.

**Supplementary Information:**

The online version contains supplementary material available at 10.1186/s13023-022-02598-x.

## Introduction

The process to a medical diagnosis is not always a straightforward, linear path. Although many diagnoses can be made by clinical examinations, bloodwork, or diagnostic imaging, for many individuals the results are inconclusive, and they do not receive a diagnosis that unifies their various symptoms and addresses their medical concerns. Reasons for referrals to pediatric genetics clinics include multiple birth defects or congenital abnormalities, intellectual disability or sensory impairment of unknown etiology, and known or suspected genetic disorders [1]. While first-tier genetic testing is informed by patient’s presenting features and the differential diagnosis, it is often a chromosomal microarray or targeted next-generation sequencing, or exome/genome sequencing [2]. Given that the diagnostic yield of these testing strategies is estimated at 40–50%, a family is likely to remain undiagnosed after this process [3, 4]. When it is suspected that there is an underlying condition, but the specific diagnosis cannot be made, these individuals are often said to have an “undiagnosed disease” or an “undiagnosed genetic condition.” While trying to obtain a diagnosis, patients may undergo many medical evaluations and tests, a process often described as “the diagnostic odyssey” [5, 6].

One program that families on a diagnostic odyssey may turn to is the Undiagnosed Disease Network (UDN). The UDN, formed in 2008, is a network of sites around the United States with a common goal to bring together clinicians and researchers to diagnose patients on a diagnostic odyssey [7, 8]. Participants who apply to the UDN have usually completed all clinically available testing and have often undergone non-diagnostic exome or genome sequencing. Families who have applied to the UDN have exhausted all other options and are extreme examples of a diagnostic odyssey [9].

Reasons families embark on a diagnostic odyssey have been well-classified and include the desire for a label, to ease navigation through the healthcare system, and a desire for diagnostic certainty [5, 10–12]. Previous studies have demonstrated that patients and families with undiagnosed diseases can have significant social, emotional, and psychological needs such as fear and anxiety about the uncertainty of the future, varying levels of stress, and lack of support [5, 12–14]. It is anticipated that parents and children will cycle through various stages of emotions, expectations, and roles throughout a diagnostic odyssey, but how and whether these factors affect decisions to continue searching for a diagnosis has not been previously explored [9, 10, 15].

Throughout the diagnostic odyssey, families of children with undiagnosed conditions must decide whether to continue pursuing a diagnosis or to end their search, accepting that they may not obtain a label for their symptoms. For families who have been on a long journey, what began as a search for a diagnosis for their young toddler may now be a search for a diagnosis for their adolescent child. Adolescence, defined as between ages 10 and 19, is an important developmental period including unique developmental milestones and changes in mentality, all focused around independence and planning for the future [16–19]. Although much is known about adolescence and the important developmental changes that occur during these formative years, little is known about the influence of an adolescent patient’s opinions in a family’s decision of whether to continue a diagnostic odyssey. This study explored the role of the adolescent in a family’s decision-making process by interviewing parents of children and adolescents who have applied to the Undiagnosed Disease Network (UDN) at the National Institutes of Health (NIH).

## Methods

### Participants and study recruitment

Study participants were parents of children who applied to the Undiagnosed Disease Network (UDN) at the National Institutes of Health (NIH) between July 2018 and January 2020. They were recruited from the pool of UDN applicants via email between October 2019 and January 2020 by a member of the UDN clinical team (EFM). Recruitment emails included basic information about the study, a link to the study survey, and the primary investigator’s contact information. All participants were sent a participation reminder via email two weeks after initial study recruitment. Participants who expressed interest in participating in an interview and provided an email address in their survey were contacted via email by a member of the study team (IM). This email included a link to a SignUpGenius.com webpage for participants to sign up for an interview.

Five dollars was donated to the Undiagnosed Disease Fund through the National Organization for Rare Disorders (NORD) for every completed survey. An additional five dollars was donated for every completed interview.

### Instrumentation

The “Supportive Care Needs” model by Pelentsov et al., which proposes a model of needs for parents of children with rare diseases, served as a framework for the development of both instruments: a screening survey and semi-structured interview guide [15]. The screening survey covered basic demographic information, including the age of the child who applied to the UDN, which parent completed the survey, At the end of the survey, participants had the option to include an email address if they wanted to be contacted for a phone interview (Additional file 1: Appendix 1).

The semi-structured interview guide explored four domains: information about the child’s condition and their history of medical examinations and testing, details of the child’s diagnostic odyssey and the family’s decision to apply to the UDN, why and whether the family has decided to continue/not continue pursuing a diagnosis for their child, and the role that healthcare providers have played in the child’s diagnostic odyssey (Additional file 1: Appendix 2).

Interviews were piloted by a member of the study team (IM) with two pediatric genetic counselors.

### Data analysis

Interviews were transcribed using REV transcription service [20]. A qualitative codebook was developed and revised with input from all members of the study team (Additional file 1: Appendix 3). The foundation of the codebook was based on Pelenstov’s Supportive Care Needs Model using the six identified needs: social, emotional, practical, physical, psychological, and informational. Subcodes to specify differences in excerpts relating to parent, child, and family were used to capture nuanced differences between the groups. Additional codes were created when topics were not well-represented by the original code book, such as codes relating specifically to the diagnostic odyssey and the health care system. The study team (IM, EFM, BY) continued to revise the codebook until all recurring interview topics had a corresponding code. All transcripts were coded by IM and at least one other member of the study team using Dedoose coding software [21]. An iterative coding approach was used until consensus in coding was reached for each interview. After consensus was reached, the codes were condensed into broader sub-themes. Discussions between team members coalesced the sub-themes into three overarching themes: (1) details of the diagnostic odyssey, (2) parental duty, and (3) tensions between parent and child.

Basic statistical analyses (means and standard deviations) were calculated in Microsoft Excel (2017). Comparative statistical analyses (chi-squared and t-tests) were calculated using GraphPad QuickCalcs (2017) to confirm that there were no continuous or categorical differences between the surveyed participant group and the interviewed participant group.

This study was approved by the University of Michigan IRB (HUM00167454).

## Results

### Demographics

One hundred four UDN applicants were recruited for the study, 50 completed the screening survey for a survey response rate of 48%. The majority (92%) of parents who completed the survey were mothers. The average age of their child was approximately eight years old, with an age range of one to eighteen years (Table 1).

**Table 1:** Participant demographics

	Total survey respondents (N = 50)	Total interviews analyzed (N = 14 parents; 13 families)
Parent surveyed		
Mother	46 (92%)	13 (93%)
Father	4 (8%)	1 (7%)
Average age of child (years) (range)	8.2 years (1–18 years)	11 years (3–18 years)
% of children ages 0–9	66%	38%
% of children ages 10–18	34%	62%
% of life spend searching for a diagnosis (range)	N/A	63% (13–100%)

Fourteen interviews, from thirteen families, were analyzed. All interviews were with parents, no children participated in the interviews. Interviews ranged in length from 22 to 53 min with an average length of 39 min. All but one of the interviewed parents were mothers (93%). The ages of the children in the interview group were slightly older (*p* = 0.068) than those in the surveyed group, with an average age of eleven years and a range of three to eighteen years. These families have spent an average of 63% of their child’s life searching for a diagnosis (Table 1).

### A diagnostic odyssey

The children of all study participants had complex medical needs that parents, and often the child themselves, had been navigating for a protracted length of time. This included evaluations by various specialists and extensive medical testing, often including genetic testing such as exome/genome sequencing.

Well, he's been to everything, genetics, neurologists and rest of the specialists, like orthopedics. He's been to [a] child psychologist… MRI, x-rays and everything. All exome sequencing and whole genome sequencing… (Participant 62)

He's been through a battery of blood tests, ultrasounds, x-rays… CT scans, MRIs, hearing tests, vision tests, brainwave tests. Pretty much everything under the sun, he's been through… (Participant 27)

She's been examined from head to toe. They've done every kind of test. So yeah, I think she's really done everything. (Participant 15)

Parents recognized that their child’s complex medical needs required them to have a high level of involvement in their child’s care and voiced ways that a diagnosis could impact this care. These reasons included ensuring that their child is receiving appropriate care, hope for future medical advances, and an overall wish for more information.

Because if he's in the hospital, then I have to cancel appointments that I have or whatever I have going on… I just make my life around his so I can make sure he's comfortable. (Participant 14)

Well, if there's something that we can be doing differently then that would be huge… so if there's something, a change of diet that would help her… to make sure there isn't something out there that would help her that we could do that would just improve her life and her future. (Participant 22)

...Advancements in medicine are being made daily… [I want] any possibility of any improvements on his life and his future. (Participant 25)

### Parental duty

Many parents described how the inherent duty of being a parent drove them to continue their diagnostic search. Parents explained that to fulfill their duty as a parent they needed to feel as though they had done everything possible and had exhausted all options before coming to the UDN.

It is my job to protect her. It's my job to make sure that she's kept alive and that she's healthy and that she has the best opportunities… And if I as her mother can’t figure out what this was, how could I expect anybody else to…? (Participant 51)

Because, really, as a parent, it's hard to see your child go through something that you can't fix. You always want to fix their boo-boo's and make them feel better. But when you can't, it makes you feel like you failed…So by finding a diagnosis, it would go a long way, I guess for me as a parent, to say, "Hey, I pushed enough that I finally found out what was wrong." And regardless of whether it can be fixed or not, it's like I pushed enough to find out why it happened and how it happened and now I know. It's peace of mind, I guess you'd say. (Participant 27)

But we just want to know what's, what else do we need to be doing, if anything. Cause I always feel like we're missing something, that we should be doing more. (Participant 26)

Parents explained that over time the way they acted on their sense of parental duty evolved. The need for a diagnosis became less of a driver, and ongoing concerns for the child’s long-term health and support is more important.

Well, because I want to help my son in the best possible way. Meanwhile, I didn't focus so much on diagnostic… Since I couldn't get a diagnosis in many years, I focused a lot on therapies and what kind of therapies could help him progress cognitively, physical strength and so on. (Participant 62)

Although some parent participants expressed that parental duty drove them to initiate and continue the diagnostic journey, others explained how it aided in the decision to pause searching. They explained that although they will always be interested in a diagnosis, their adaptation to their child’s condition had led to a shift in their priorities, and their energy is now focused on providing the best care for the child, rather than split between care and searching for a diagnosis. They have reached acceptance and understanding that a diagnosis may not be feasible. Importantly, most parents explained that although the need for a label may no longer be their primary focus, they are not interested in “giving up” and will continue to pursue additional diagnostic options as they are presented.

I'm not desperate in my search for a diagnosis, so it's not a matter of stopping. We're always open for anything… I'm not so anxious to get a diagnosis, but I certainly will never stop as long as there's something to look at. (Participant 35)

### Parent and child/adolescent opinions may conflict and cause tension

The lived experience of parent and child are one in the same at the start of the diagnostic odyssey; young children have limited agency and experience their parents’ journey as their own. However, over time, the child’s needs and perspectives diverge and may result in conflict with those of their parents. This tension may be exacerbated as the child enters adolescence and moves towards adulthood and possible independence.

At one point, when he was younger, I do think we were driven by more of the frustration of, ‘How can there not be anything wrong with him?’ And we need to keep searching and we need to keep looking… So, I do think that did drive us to keep looking. Now, with his negative frustration and negative emotions about, "Why can't anybody fix this? Why can't anybody figure out what's wrong with me?" That has kind of come into play in the past year or two… I don't feel like I am driven as much anymore by the negatives that I was initially, but now I have his negative emotions driving it, because he's older. (Participant 18)

With the wish by the adolescent for independence can come a desire for an increased role in decision-making, including decisions about their own healthcare. Understanding that the adolescent may eventually need to take more control of their healthcare and life decisions is a contributing factor to why families continue searching for a diagnosis.

I ended up pulling him out of school and homeschooling him to see if we could protect him from all the germs of being at school… I had a child that longed for nothing more than to be at school with his friends. That's all he wanted. So, emotionally, it was not the right choice even though he was healthier. He begged to go back. Then told me he'd rather spend every other month sick in the hospital than stay at home away from his friends, so I had to respect that. (Participant 18)

My feeling is I want my daughter to try the [specialized] program relating to [symptoms]…So I'm kind of in a hold pattern now, waiting for her to agree to go to this program and see if that treatment helps her…She just refuses to go. She's 16 years old. I can't force her to do something she doesn't want to do. (Participant 37)

Parents noted that their children are now spending time planning their future and they may not consider how their complex medical needs may alter this imagined future. Children had anticipated plans for their teenage and adult years, but what was imagined may no longer be plausible. Misalignment of goals occurs while adolescents balance this disappointment and create new opportunities for the future, while parents navigate their revised role in their child’s uncertain future.

As far as his personal growth is concerned, it's been quite a setback. He's 16 now, he was 14 when he lost his vision. He was just starting to become a teenager and now he is a teenager, and he has a couple of really good friends. But a lot of what he had planned for his teenage years isn't happening... (Participant 27)

I feel like we're living two different lives. One, I am letting him socially talk about going to college and being a very typical 17-year-old and filling out applications just like his friends are doing, but the reality is he's not well enough to go. We're going to have to figure that piece out. (Participant 18)

He already has some familiarity with "look you can be blind and an oceanographer, you can be blind and a parent, you can be blind and a teacher." Your brain will work around that. So your future doesn't have to be limited. But we don't really know if it's just his eyes. We don't know if there's something with the brain or what will happen if he hit adolescence and adulthood. (Participant 58)

## Discussion

Interviews with fourteen parents of children with undiagnosed diseases at various stages of their diagnostic journey helped to understand motivating factors for “why” families continue to search for a diagnosis after years of inconclusive testing. As a child matures, differences between parent and child opinions about the diagnostic search may conflict, often serving as a re-evaluation point for family decision-making of whether to continue the odyssey. Parent(s) and adolescent may have differing views of the obtainability of a child’s autonomy, and the idea of whether to keep future options open or take a more “realistic” approach can create inter-familial tension. Additionally, the innate parental drive may encourage families to continue a search, but this parental drive is tempered by the emerging voice of the adolescent. Whether and how the family incorporates the developing independent voice of the child will change the way decision-making regarding the odyssey occurs within the parent/child dyad (triad). These factors work in combination to help families decide whether, and when, they should continue to search for a diagnosis.

The developing strength of the child’s voice, through the eyes of the parent, was a major theme as both a motivator and barrier of the diagnostic odyssey. The way in which parents discussed their children, most in their adolescent years, shed unique light on how the parent/child tension impacts the decision to continue the odyssey. Adolescence is often thought of as the second individualization crisis, and involves multiple physical, psychological, sexual, and social changes [16, 18, 22]. It is normal and anticipated that children at this age are starting to want autonomy and independence from their parents, but when a child has complex medical needs and depends on their parents for critical medical care, there is an additional layer of tension between the parents and adolescent. The adolescent may feel ready to create distance between themself and their parents, moving towards independence and taking charge of their own medical care, but this may not always be realistic or feasible in children with rare/complex conditions.

The parental narrative emphasized the emerging voice of their child, highlighting the dynamic balance between parental and child opinions on a medical journey. Parent participants emphasized that their child’s voice is not replacing theirs; rather, there is a need to incorporate one into the other to determine the best path forward for the family. A major difference between the parental and child’s experience is the distinction between an odyssey and a journey. The children, moving from following their parents’ direction to making decisions about their own medical experience and forming individualized opinions, are on a journey: a passage or process from one stage to another [23]. The parents, having navigated their child’s medical experience for an extended period of time often with multiple notable decisions and possible hardships, have moved from being on a diagnostic journey to a diagnostic odyssey [24, 25]. This distinction helps clarify why adolescence can be a point of reinvigoration or a cessation of searching; children are now driving their own search as they make sense of their diagnostic journey.

Keeping a child’s future options open until they reach an age of autonomy to make their own decisions, an “open future”, is not always realistic for an individual with complex medical needs and differences in expectations can create tension between parent and child. Participants in this study highlighted the tension between their opinions and their child’s, giving examples such as the child’s desire for romantic relationships, plans for higher education, and career goals. When complex medical needs are involved, the adolescent’s plans may not be possible or may conflict with what parents perceive as realistic [16–18]. The idea of an open future may still exist for an adolescent who is earlier in their medical journey and has not yet fully understood their limitations. This can lead to additional levels of tension when the parents know that their child’s imagined future may not be plausible. These tensions can be further exacerbated when an individual does not have a formal diagnosis and their prognosis is unknown, leading many parents to continue the search for a diagnosis to better support their children in “realistic” plans for their future.

Parents must balance continuing the diagnostic odyssey with whether to allow their child to take control of medical decision-making. Allowing the child to have more input allows for greater autonomy but can potentially seem like a pause or a step backwards in the parents’ diagnostic odyssey timeline. Our novel insight into the relationships between parent and child revealed that as time passes in the diagnostic odyssey, parents may shift their mindset about the goal of their odyssey, from working towards a diagnosis to understanding how to help their child live their best, most fulfilling life. Simultaneously as parents are moving towards acceptance of having their child remain undiagnosed, the child matures and moves towards adolescence and adulthood, often forming their own opinions about pursuing a diagnosis. The child will experience emotional aspects of the diagnostic odyssey that their parents experienced years before; both parents and child are experiencing similar thoughts and transitions about the diagnostic odyssey but are experiencing them years apart.

It is important for both families and healthcare professions to understand that the decision about whether to continue a diagnostic odyssey is not static; it is expected to be dynamic and constantly evolving. The child’s journey may follow a different trajectory than the parent’s, creating multi-dimensional levels of tension between them. These tensions can cause conflict and stress, but also help a family make important decisions about the continuation of a diagnostic odyssey. This tension exists both internally (within a parent’s own thoughts and emotions) and externally (between parent and child). It also transpires across multiple domains, including the shifting drive of parental duty, the child’s potentially unobtainable desire for autonomy, and managing the child’s idea of an open future. These interviews brought to light how the milestone of adolescence can be a “check point” for a family to reevaluate whether to continue the diagnostic odyssey. At this check point, adolescent voices are emerging and may change the trajectory of the family’s odyssey. It is crucial that healthcare providers, such as genetic counselors, understand both patients’ and parents’ motivations driving their desire to continue or halt the diagnostic odyssey to best support a family’s decision-making process and provide appropriate patient-centered care. Revisiting conversations that may have been had years prior, such as why a family desires a diagnosis, may be helpful at this stage and may help a family understand how to incorporate opinions that previously did not have a voice into decision-making. A decision to stop the diagnostic odyssey made when the child was five years old may be reconsidered years later, when the child wants their voice and opinion to be heard.

## Conclusion

This study investigated how and why a family decides to continue, stop, or pause a diagnostic odyssey. We found that a child’s entrance into adolescence can serve as a check point on a diagnostic search, forcing parents to decide if and how to include a child in medical decision-making. Parental narratives served as a proxy for their child’s voice, rather than the child’s voice themselves given that many of the children of interviewed parents have cognitive impairments of varying degrees and are unable to engage in an interview with a stranger in a formal setting. There is the need for ongoing conversations between providers and families about the goals of continuing a search, making sure to include the child in an age and developmentally appropriate manner.

## Supplementary Information

**Additional file 1**. Study survey and semi-structured interview guides.

## Data Availability

The datasets generated and analyzed during the current study are available from the corresponding author on reasonable request.
