# Supplementary material for: Continuing a search for a diagnosis: the impact of adolescence and family dynamics
Source: Orphanet J Rare Dis. 2023 Jan 9;18:6. doi: 10.1186/s13023-022-02598-x (PMC9830697; doi:10.1186/s13023-022-02598-x)
Supplement: Supplementary file 1 — Additional file 1. Study survey and semi-structured interview guides. [file 13023_2022_2598_MOESM1_ESM.docx]

**Appendix 1**

**UDN and University of Michigan Collaboration Survey**

Thank you for taking our survey. This survey explores your experiences caring for your child with an undiagnosed condition. This study, “Continuing a Search for a Diagnosis: Perspectives from Parents of Children with Undiagnosed Diseases,” aims to understand why families continue to search for a diagnosis for their child. The study has been approved by the University of Michigan IRB (HUM00167454).

Participating in this study is entirely voluntary. Participating will have no effect on your UDN involvement, including decisions regarding your application, or on your ongoing clinical care. We at the University of Michigan do not have access to your clinical or any personal information you provided to the UDN. You may stop the survey at any time. There are no risks in participating in this study.

At the end of the survey is a question asking about your interest in a follow-up interview. We aim to interview approximately 30 participants. If you decide not to be interviewed, or if we are unable to interview you, your deidentified data will still be part of our survey data.

If you have questions about the survey or participating in this study, please contact Ilana Miller at [email address].

By continuing to the next page of the survey, you provide your consent to participate in the study.

Q1 Are you a mother or father of a child with an undiagnosed condition?

- Mother
- Father
- Other: ________________________________________________

Q2 How old (in years) is your child with an undiagnosed condition?

________________________________________________________________

Q3 Which of the following describes your involvement with the Undiagnosed Disease Network (UDN)?

- We have applied to the UDN and are waiting to hear back
- We have been accepted into the UDN but have not yet had our appointment at the National Institutes of Health
- We were not accepted into the UDN
- Other: ________________________________________________

Q4 Do you think there is a genetic cause/reason for your child's condition?

- Yes
- No
- Uncertain

Q5 Please think about your experiences caring for your child with an undiagnosed condition and indicate your agreement with the following statements.

|  | Agree | Disagree |
| --- | --- | --- |
| Having more information about how to care for my child's health needs would NOT be helpful to me |  |  |
| I experience fewer positive/pleasant emotions and feelings since caring for my child's undiagnosed condition (examples: happiness/joy, excitement, relaxedness) |  |  |
| Due to caring for my sick child, my own health care needs are not taken care of (examples: not getting enough sleep, frequent illness, weight loss or gain) |  |  |
| I need additional support caring for my child on a day-to-day basis (examples: childcare, transportation, paying bills) |  |  |
| I experience negative/unpleasant emotions or feelings related to my child's undiagnosed condition (examples: anger, frustration, anxiety) |  |  |
| I DON’T have trouble finding information (online, in books, from health care providers, etc.) about how to care for my child |  |  |
| Dealing with my child's undiagnosed conditon makes me feel lonely and isolated from others, such as my friends and other family members |  |  |
| I view my relationship(s) with my child's health care provider(s) as positive and helpful |  |  |

Q6 We are interested in learning more about your experiences with your child's undiagnosed condition. If you are willing to be contacted about a follow-up interview to further discuss this topic, please leave your email address below.

________________________________________________________________

Q7 What is your name?

________________________________________________________________

**Appendix 2**

**INTERVIEW GUIDE- Group 1 (accepted to UDN) and Group 2 (applied to UDN)**

Thank you for agreeing to participate in this interview. The goal for conducting this interview is to follow-up on your survey responses and to learn more about why a family decides to continue or stop searching for a diagnosis. This interview is part of our larger study titled “Continuing a Search for a Diagnosis: Perspectives from Parents of Children with Undiagnosed Diseases.” The study has been approved by the University of Michigan IRB (HUM00167454).

Participating in this study is entirely voluntary. Participating will have no effect on your UDN involvement, including decisions regarding your application, or on your ongoing clinical care. We at the University of Michigan do not have access to your clinical or personal information that you have provided through the UDN.

We anticipate that the interview will take 30-45 minutes. The interview is being recorded. You may stop the interview at any time. You do not have to answer any questions that you do not want to answer. There are no risks in participating in this study. Your responses will be de-identified before we analyze the data.

By agreeing to continue with the interview, you are giving your informed consent to participate.

| Condition history/initial assessment | Can you tell me about the top 3 aspects of your child’s condition that interfere with day to day life? | What are the top 3 symptoms that your child has? | |
| --- | --- | --- | --- |
|  | How many years have you been searching for a diagnosis? | How old was your son/daughter when you started noticing symptoms/that something wasn’t right? | |
|  | What different kinds of evaluations has your child had? | What providers/specialists does he/she see? | |
|  |  | Has your child been diagnosed with any conditions or diseases? | |
|  | Tell me about your support system/community at home | Who knows about your search for your child’s diagnosis? | |
|  | I saw that you marked that you (think that there is)/(think that there isn’t)/(are unsure if there’s) a genetic cause to your child’s disease. Can you tell me more about why you think this? | Has he/she had any genetic testing? | What kind of genetic testing has he/she had? |
|  |  |  | What were the results of the genetic testing? |
|  |  | Is there anyone else in the family that has similar features as your child? | |
| Diagnostic Odyssey/UDN | It sounds like it’s been a long search for a diagnosis, and your child has had multiple evaluations and undergone many different tests without receiving an answer or a diagnosis. Can you tell me about your journey to the Undiagnosed Disease Network (UDN) and what this search has been like as a parent? | Why did you decide to participate in the UDN? | What are you hoping to gain from participating in the program? |
| Decision to continue to pursue a diagnosis | Can you tell me about why you’ve decided to continue to pursue a diagnosis for your child after going through this diagnostic odyssey? | How do you think a diagnosis will change your child’s health care? | |
|  |  | How do you think a diagnosis will affect other aspects of your/your child’s life? | |
|  | What were the major motivating factors that made you decide to continue pursuing a diagnosis for your child? | I see that in your survey you said that you [incorporate survey responses that indicate no additional need for support]. Can you tell me more about this? | How important is this in caring for your child? |
|  |  |  | If you didn’t have this support, would this affect your ability to continue to search for a diagnosis for your child? |
|  |  | You said that you [incorporate survey responses that indicate an additional need for support]. Can you tell me more about this? | If you had this support, do you think it would affect your ability to care for your child and to continue to search for a diagnosis? |
|  | Have you ever thought about stopping your search for a diagnosis? | *If yes-* Can you tell me about what this time in your life looked like? | What do you think motivated you to consider stopping? |
|  |  |  | Where there any big changes going on in your life? |
|  |  |  | Did you learn anything new about your child’s health that you think impacted this thought to stop pursing a diagnosis? |
|  |  |  | What made you change your mind and decide to continue pursuing a diagnosis? |
|  |  | *If no-* can you envision anything that could impact your decision to continue pursuing a diagnosis? | Has anyone ever pressured you to stop? |
| Role of HCPs | Earlier we talked about the different providers and specialists that your child sees. Do you view one of these providers as your child’s primary provider? | Can you tell me about your relationship with this provider? | How has this provider supported you, or not supported you, in your decision to continue pursuing a diagnosis for your child? |
|  |  | Is there anything that any of your health care providers have done that helped you decide to continue/stop continuing to pursue a diagnosis? | Is there anything that you wish they did do or didn’t do? |
|  |  |  | Overall, have your child’s health care providers been supportive/helpful in your journey to a diagnosis? |
| When you think about our conversation, have you told me everything that you want our team to know about your family’s decision to continue/stop pursing a diagnosis for your child? | | | |

**INTERVIEW GUIDE- Group 3 (not accepted to UDN)**

Thank you for agreeing to participate in this interview. The goal for conducting this interview is to follow-up on your survey responses and to learn more about why a family decides to continue or stop searching for a diagnosis. This interview is part of our larger study titled “Continuing a Search for a Diagnosis: Perspectives from Parents of Children with Undiagnosed Diseases.” The study has been approved by the University of Michigan IRB (HUM00167454).

Participating in this study is entirely voluntary. Participating will have no effect on your UDN involvement, including decisions regarding your application, or on your ongoing clinical care. We at the University of Michigan do not have access to your clinical or personal information that you have provided through the UDN.

We anticipate that the interview will take 30-45 minutes. The interview is being recorded. You may stop the interview at any time. You do not have to answer any questions that you do not want to answer. There are no risks in participating in this study. Your responses will be de-identified before we analyze the data.

By agreeing to continue with the interview, you are giving your informed consent to participate.

| Details of condition/ genetic cause | Can you tell me about the top 3 aspects of your child’s condition that interfere with day to day life? | What are the top 3 symptoms that your child has? | |
| --- | --- | --- | --- |
|  | How many years have you been searching for a diagnosis? | How old was your son/daughter when you started noticing symptoms/that something wasn’t right? | |
|  | What different kinds of evaluations has your child had? | What providers/specialists does he/she see? | |
|  |  | Has your child been diagnosed with any conditions or diseases? | |
|  | Tell me about your support system/community at home | Who knows about your search for your child’s diagnosis? | |
|  | I saw that you marked that you (think that there is)/(think that there isn’t)/(are unsure if there’s) a genetic cause to your child’s disease. Can you tell me more about why you think this? | Has he/she had any genetic testing? | What kind of genetic testing has he/she had? |
|  |  |  | What were the results of the genetic testing? |
|  |  | Is there anyone else in the family that has similar features as your child? | |
| Diagnostic Odyssey/UDN | It sounds like it’s been a long search for a diagnosis, and your child has had multiple evaluations and undergone many different tests without receiving an answer or a diagnosis. Can you tell me about your journey to the Undiagnosed Disease Network (UDN) and what this search has been like as a parent? | Why did you decide to participate in the UDN? | What are you hoping to gain from participating in the program? |
| Decision to continue/stop pursing a diagnosis | I know your child was not accepted into the UDN. Have you thought about if you’re still going to continue searching for a diagnosis? | Can you tell me about how you made this decision? | |
|  | *If continuing to search for diagnosis* | How do you think a diagnosis will change your child’s health care? | How do you think a diagnosis will affect other aspects of your/your child’s life? |
|  | *If stopping to search for diagnosis* | How do you thinking stopping to pursue a diagnosis will impact your child’s health care, if at all? | How do you think stopping to pursue a diagnosis will affect other aspects of your/your child’s life, if at all? |
|  | What were the major motivating factors that made you decide to continue/stop pursuing a diagnosis for your child? | I see that in your survey you said that you [incorporate survey responses that indicate no additional need for support]. Can you tell me more about this? | How important is this in caring for your child? |
|  |  |  | If you didn’t have this support, would this affect your decision to continue/stop to search for a diagnosis for your child? |
|  |  | You said that you [incorporate survey responses that indicate an additional need for support]. Can you tell me more about this? | If you had this support, do you think it would affect your decision to care for your child and would impact your decision to continue/stop pursuing a diagnosis? |
|  | Before applying to the UDN, had you ever thought about stopping your search for a diagnosis? | *If yes-* Can you tell me about what this time in your life looked like? | What do you think motivated you to consider stopping? |
|  |  |  | Where there any big changes going on in your life? |
|  |  |  | Did you learn anything new about your child’s health that you think impacted this thought to stop pursing a diagnosis? |
|  |  |  | What made you change your mind and decide to continue pursuing a diagnosis? |
|  |  | *If no-* can you envision anything that would have impacted your decision at the time to continue pursuing a diagnosis? | Has anyone ever pressured you to stop? |
| Role of HCPs | Earlier we talked about the different providers and specialists that your child sees. Do you view one of these providers as your child’s primary provider? | Can you tell me about your relationship with this provider? | How has this provider supported you, or not supported you, in your journey to pursue a diagnosis for your child? |
|  |  | Is there anything that any of your health care providers have done that helped you decide to continue/stop continuing to pursue a diagnosis? | Is there anything that you wish they did do or didn’t do? |
|  |  |  | Overall, have your child’s health care providers been supportive/helpful in your journey to a diagnosis? |
| When you think about our conversation, have you told me everything that you want our team to know about your family’s decision to continue/stop pursing a diagnosis for your child? | | | |

**Appendix 3**

| **Codes** | | | **Description** |
| --- | --- | --- | --- |
| Relationship w/ HCP | | | Impact of various health care providers on patient/parent/family’s diagnostic odyssey, decisions about healthcare, how they view the medical system, etc. |
| Family support | Parent | | Presence or absence of family support |
|  | Child | |  |
| Friends support | Family | | Presence or absence of friend support |
|  | Parent | |  |
|  | Child | |  |
| Spousal support | | | Presence or absence of family support or any aspect of the relationship between the parent and his/her spouse |
| Support groups | Family | | Involvement or mention of support groups; positive or negative |
|  | Parent | |  |
|  | Child | |  |
| Parent/family/child relationship | | | Description or mention of relationship or tension between either parent, the family, and/or the affected child; positive or negative |
| Emotional/psychological needs  (positive or negative) | Stress/  anxiety/  depression | Family | Direct mention of, or statements expressing, stress, anxiety, and/or depression |
|  |  | Parent |  |
|  |  | Child |  |
|  | Guilt/blame | Family | Direct mention of, or statements expressing, guilt and/or blame |
|  |  | Parent |  |
|  |  | Child |  |
|  | Isolation/loneliness | Family | Direct mention of, or statements expressing, isolation or loneliness (presence of absence) |
|  |  | Parent |  |
|  |  | Child |  |
|  | Fear | Family | Direct mention of, or statements expressing, fear |
|  |  | Parent |  |
|  |  | Child |  |
|  | Trust/distrust | Family | Direct mention of, or statements expressing, trust or distrust (to medical system, treatment options, possible diagnoses, etc.) |
|  |  | Parent |  |
|  |  | Child |  |
|  | Anger | Family | Direct mention of, or statements expressing, anger |
|  |  | Parent |  |
|  |  | Child |  |
|  | Frustration | Family | Direct mention of, or statements expressing, frustration |
|  |  | Parent |  |
|  |  | Child |  |
|  | Coping | Family | Direct mention of, or statements expressing, coping and coping techniques |
|  |  | Parent |  |
|  |  | Child |  |
|  | Self-efficacy/empowerment | Family | Direct mention of, or statements expressing, self-efficacy or empowerment |
|  |  | Parent |  |
|  |  | Child |  |
|  | Validation | Family | Direct mention of, or statements expressing, the lack of, or need for validation |
|  |  | Parent |  |
|  |  | Child |  |
|  | Unspecified emotional/psychological needs | Family | Direct mention of, or statements expressing, an emotional or psychological need that does not fall into another category |
|  |  | Parent |  |
|  |  | Child |  |
| Parental duty | | | Direct mention of, or statements expressing, the parent’s felt parental duty to his/her child |
| Financial concerns/parental job | | | Insurance issues, impact on work/career, other discussions regarding finances; positive or negative |
| Educational needs | Family | | Desire for additional education needs; not needing any education regarding the child’s condition |
|  | Parent | |  |
|  | Child | |  |
| Unaccepting of explanations | Family | | Direct mention of, or statements expressing the family/parent/child’s inability to accept possible explanations for the child’s health condition |
|  | Parent | |  |
|  | Child | |  |
| Wanting a label | Family | | Direct mention of, or statements expressing, the desire to have a label for the child’s health condition |
|  | Parent | |  |
|  | Child | |  |
| Idea of being stuck | Family | | Direct mention of, or statements about being “stuck” regarding next steps in pursuing a diagnosis |
|  | Parent | |  |
|  | Child | |  |
| Health care system motivators/barriers | | | Long wait times to get into clinic, referral issues, logistics regarding clinics or the greater health system, accessing services |
| Genetic cause | | | Thoughts about whether there is or is not a genetic cause to the child’s condition |
| Openness | Family | | Openness to disclosing child’s health information, information about their diagnostic odyssey, etc. |
|  | Parent | |  |
|  | Child | |  |
| Imagined impact of having/not having a diagnosis | | | How having/not having a diagnosis will impact aspects of the child’s life (ex: losing hope) |
| Uncertain future | Family | | Direct mention of, or statements expressing, uncertainty (prognosis, management, treatment, career, education, etc.) |
|  | Parent | |  |
|  | Child | |  |
| Drivers of the diagnostic odyssey | | | Motivations for deciding to continue/stop the diagnostic odyssey (important timepoints or events in the child’s diagnostic odyssey; risk to future generations; etc.) |
| Disease/symptom management/treatment | | | Discussion of current or future disease/symptom management or treatment |
| Acceptance of no diagnosis | | | Indirect or direct expression of accepting that there may not be an explanation for the child’s condition |
| Hiatus/temporary pause in search | | | Any period of time where they made the decision to stop pursuing a diagnosis and/or explanation of why this decision was made |
| Impact of family history | | | Any mention of how a presence or lack of family history of a related or unrelated family history of disease has impacted the diagnostic odyssey or how they think about the child’s condition |
| Misc. code | | | Excerpt that should be coded but unsure which code it belongs in |
